# Additional Blue LED during Cultivation Induces Cold Tolerance in Tomato Fruit but Only to an Optimum

**DOI:** 10.3390/biology11010101

**Published:** 2022-01-09

**Authors:** Fahrizal Yusuf Affandi, Teddy Prayoga, Theoharis Ouzounis, Habtamu Giday, Julian C. Verdonk, Ernst J. Woltering, Rob E. Schouten

**Affiliations:** 1 Horticulture and Product Physiology, Wageningen University and Research, P.O. Box 16, 6700 AA Wageningen, The Netherlands; teddy.prayoga@outlook.com (T.P.); T.Ouzounis@osram.com (T.O.); habtamu.gebraegziabher@infarm.com (H.G.); julian.verdonk@wur.nl (J.C.V.); ernst.woltering@wur.nl (E.J.W.);; 2 Bioresource Technology and Veterinary Department, Vocational College, Universitas Gadjah Mada, Yogyakarta 55281, Indonesia; 3 Food & Biobased Research, Wageningen University and Research, P.O. Box 17, 6700 AA Wageningen, The Netherlands

**Keywords:** chilling injury, tomato fruit, lycopene, blue LED

## Abstract

**Simple Summary:**

LED lighting is increasingly applied to increase yield and quality of greenhouse produced crops, especially tomatoes. Tomatoes cannot be stored at cold temperatures due to chilling injury that manifests as quick quality deterioration during shelf life. The aim of this study is to investigate whether additional blue LED lighting can mitigate the negative effects of cold storage for ‘Foundation’ tomatoes. We applied three treatments, 0, 12 or 24% additional blue light during cultivation, and investigated quality attributes at harvest, after cold storage and subsequent shelf-life. We observed that red harvested tomatoes cultivated with 12% additional blue light acquired cold tolerance. Interestingly, these tomatoes were slightly less red colored at harvest and showed a faster loss of red color during cold storage. The measured red color is closely related to the lycopene concentration. We hypothesize that lycopene, a known antioxidant, present in 12% additional blue cultivated tomatoes mitigates chilling injury. Other antioxidants present in tomatoes were only affected by the ripeness at harvest and were therefore not involved in the acquired cold tolerance. The cultivation of tomatoes using additional blue LED is an attractive way to produce tomatoes that can withstand long transport at cold temperatures at the expense of a slightly less red tomato at the consumer.

**Abstract:**

Tomato is a chilling-sensitive fruit. The aim of this study is to examine the role of preharvest blue LED lighting (BL) to induce cold tolerance in ‘Foundation’ tomatoes. Blue and red supplemental LED light was applied to achieve either 0, 12 or 24% additional BL (0B, 12B and 24B). Mature green (MG) or red (R) tomatoes were harvested and cold stored at 4 °C for 0, 5, 10, 15 and 20 d, and then stored for 20 d at 20 °C (shelf life). Chilling injury (CI) indices, color and firmness, hydrogen peroxide, malondialdehyde, ascorbic acid and catalase activity were characterized. At harvest, R tomatoes cultivated at 12B were firmer and showed less coloration compared to fruit of other treatments. These fruits also showed higher loss of red color during cold storage and lower CI symptoms during shelf-life. MG tomatoes cultivated at 12B showed delayed coloring (non-chilled) and decreased weight loss (long cold stored) during shelf life compared to fruit in the other treatments. No effects of light treatments, both for MG and R tomatoes, were observed for the selected antioxidant capacity indicators. Improved cold tolerance for R tomatoes cultivated at 12B points to lycopene having higher scavenging activity at lower concentrations to mitigate chilling injury.

## 1. Introduction

Tomato (*Solanum lycopersicum* L.) is one of the most popular consumer fruits often stored at low temperature to extend shelf life [1]. Unknown to many consumers and producers, tomato is a cold-sensitive fruit that suffers from chilling injury (CI) [2]. Exposure to temperatures below 12 °C followed by storage at higher temperatures will result in reduced keeping quality, reduced flavor life and negative consumer appreciation [3,4]. CI is caused by a sequence of events, starting with an increase in cell membrane micro viscosity, followed by increases in reactive oxygen species (ROS) causing further membrane malfunctioning (lipid peroxidation), protein oxidation, enzymatic activity inhibition and, finally, damage occurring to DNA and RNA [5,6,7]. CI symptoms in tomato fruit include the inability to ripen (lack of lycopene synthesis and production of unfavorable volatiles) and accelerated decay (firmness loss, susceptibility to pathogens and water soaking), which reduces consumer acceptability [8]. The effect of chilling on the activities of cell wall degradation enzymes is not very clear [9]. Chilling reduced the activity of PG, β-galactosidase and pectate lyase (PL), but not PME [10]. Enhanced softening during the shelf life of tomatoes was not correlated with PG activity; instead, it was associated with PME activity [11]. However, Rugkong et al. [12] did not find that cold storage retarded PME activity in tomato. Cold tolerance in tomato is mainly determined by the antioxidant capacity [13,14,15]. The extent of oxidative stress can be indicated by the level of malondialdehyde (MDA), which is a product of membrane lipid peroxidation [16]. Ascorbic acid (AsA) and catalase (CAT) are known to scavenge H_2_O_2_, a major ROS with a long half-life [17,18]. One of tomato CI symptoms is lycopene degradation in red ripe tomato, which does not only reduce tomato nutritional value, but also decreases its visual quality [19,20]. Furthermore, lycopene is considered the most efficient quencher of ROS among carotenoids [21,22].

Moderate stress, such as temperature, water status or light during cultivation, trigger plants to react by initiating immediate protection against the stressor. As a result to the initial stressor, the plant will develop a certain defence mechanism to confer protection against other stresses simultaneously or subsequently [23,24,25]. For example, water deficit induces production of abscisic acid (ABA) and increases antioxidant levels for superoxide dismutase, CAT, ascorbate peroxidase, and glutathione reductase [26,27,28]. Water deficit also induces dehydrin production, proteins that are suggested to have protective effects against water and temperature stress [29]. Dehydrin genes are expressed under the regulation of ABA-dependent and ABA-independent signaling pathways [30,31]. Cold tolerance can also be facilitated by the accumulation of cryo-protectants, such as soluble sugars, sugar alcohols and amino-acid-derived compounds, including proline and glycine betaine, and activated antioxidant defence system [32].

Recently, the role of far-red LED lighting during cultivation to induce chilling tolerance was examined. In prior to being long cold stored MG-tomatoes cultivated with additional far-red LED light, reduced weight loss, less pitting and faster red color development during shelf life was observed. Red harvested tomatoes, cultivated with additional far-red light were firmer at harvest, showed reduced weight loss and less decay during shelf life after prior cold storage [33]. Far-red LED light (non-photosynthetically active radiation) during cultivation therefore induces postharvest cold tolerance in tomato. Far- red might affect the expression of the C-repeat-binding factors (CBF) genes that regulate the expression of cold responsive (COR) genes leading to membranes stabilization during cold stress [34,35]. Heat tolerance is provided by production of heat shock protein that is known to also provide protection against cold stress [13,36]. However, more information is needed to elucidate whether far-red is also able to induce production of heat shock proteins.

Our aim is to investigate whether photosynthetically active radiation during cultivation is also able to induce chilling tolerance. In strawberry, blue light (BL) illumination during postharvest storage increased the activity of antioxidant enzymes as well as the content of antioxidants, such as AsA and tocopherol [37]. We hypothesized that the addition of BL during tomato cultivation induces higher antioxidant capacity to protect tomato fruit against chilling-induced oxidative stress. We show that supplemental BL light, up to an optimum, induced chilling tolerance in red harvested tomatoes.

## 2. Materials and Methods

### 2.1. Plant Material and Growth Conditions

Tomato (*Solanum lycopersicum* ‘Foundation’) seeds were sown on 20 November 2016. Fourteen days after germination, uniform seedlings were transplanted. On 10 February, 43 d after sowing, plants (34 cm tall) were transferred to the experimental glasshouse compartment of Wageningen University, the Netherlands, and treatments started. Plants were grown on rockwool slabs in a double row ‘high wire’ system. Climate set points were as follows: temperature 22/16 °C (day/night) and a relative humidity of 78%. The plants were irrigated with a nutrient solution (12.4 mM NO_3_^−^, 7.2 mM K^+^, 4.1 mM Ca^2+^, 3.3 mM SO_4_^2−^, 1.8 mM Mg^2+^, 1.2 mM NH_4_^+^, 1.1 mM PO_4_^3+^, 30 µM BO_3_^3+^, 25 µM Fe^3+^, 10 µM Mn^2+^, 5 µM Zn^2+^, 0.75 µM Cu^+^, and 0.5 µM MoO_4_^2+^; Yara Benelux B.V., Vlaardingen, The Netherlands). Electrical conductivity (2.1 dS m^−1^) and pH (5.5) of the irrigation solution were monitored and adjusted daily. Further details of the greenhouse management are described in Kaiser et al. [38].

### 2.2. Light Treatments

Three combinations of blue and red supplemental light were obtained by combining several LED light sources, resulting in 0, 12 and 24% additional BL in a red-light background (referred to as 0B, 12B, and 24B), while keeping the total photosynthetically active radiation constant. Overhead supplemental lighting was provided by Greenpower PM-B150LO, Greenpower TL-DRBLBHO and Greenpower TLDRBMBHO modules (Philips, Eindhoven, the Netherlands) and intracanopy lighting was provided by Greenpower PMB150LO, Greenpower PM-DR150 and Greenpower interlighting DR/B modules (Philips). Once plants reached a threshold distance below overhead LED (38 cm), their stems were lowered weekly to keep their apices at a constant distance from the lamps. The spectral output of overhead and intracanopy lamps can be found in [38]. Overhead LEDs supplied 123 µmol photons m^−2^ s^−1^ at reference height. The light intensity of overhead lamps and sunlight decreased exponentially as a function of plant height and was adapted using an exponential function as detailed in Kaiser et al. (2019). Intracanopy maximum light intensity was 86 µmol m^−2^ s^−1^ measured at 75 cm distance from the lamps using a 1 m long line quantum sensor (LI-191SA, LI-COR Biosciences, Lincoln, NE, USA). On average, sunlight contributed 54%, overhead lighting contributed 31.5% and intracanopy lighting contributed 14.5% of the total daily light integral. The photoperiod was 16 h. That is, overhead and intracanopy lamps were switched on 16 h before sunset and switched off at sunset. Additionally, lamps were switched off when global radiation outside the greenhouse exceeded 450 W m^−2^ and switched on when below 250 W m^−2^. All side walls of the greenhouse compartment were closed off using a reflective screen, to prevent light pollution from neighboring compartments. Light treatments were separated by white/black/white double plastic screens.

### 2.3. Fruit Selection, Storage Conditions and Sample Preparation

Sufficiently large and uniform colored mature green (MG) and red (R) tomato fruits from all light treatments were harvested on 19 June 2017. The average weight of MG and R tomatoes was 137 ± 79 and 169 ± 47 g, respectively. Directly after harvest, tomatoes were randomly assigned for destructive or non-destructive analysis. The effect of the light treatments at harvest was characterized by randomly selecting 25 MG and 25 R-tomatoes per light treatment. Five randomly assigned tomatoes per light treatment and maturity stage were taken for non-destructive measurements during shelf life after dark storage for either 0, 5, 10, 15 or 20 d at 4 °C, in total 75 MG and 75 R tomatoes. Five randomly assigned tomatoes per light treatment and maturity stage were taken for destructive analysis, four times during cold storage. Tomatoes were dark stored for either 0, 5, 10 or 20 d at 4 °C, in total 240 MG and 240 R tomatoes.

All tomatoes were marked on two positions on the equator for repeated color and firmness measurements over time on the same tomatoes approximately every two days during shelf life. In addition, fresh weight and three chilling indices were assessed approximately at the same interval. Individual fruits, assigned for destructive measurements, were cut into small pieces and quickly frozen in liquid nitrogen and later ground to a fine powder for chemical analyses.

### 2.4. Color and Firmness Measurements

Color was assessed non-destructively by a hand-held photodiode array spectrophotometer (Pigment Analyzer PA1101, CP, Germany). Remittance was assessed at 570 (*R_570_*) and 780 (*R_780_*) nm by calculating the normalized anthocyanin index (*NAI*) (Equation (1)), which is normalized value between −1 and 1 [39]:

(1) $$NAI=\frac{R_{780}-R_{570}}{R_{780}+R_{570}}$$

Non-destructive stiffness was measured using a commercial acoustic firmness tester (AFS, AWETA, Nootdorp, The Netherlands) with the tick power of the plunger set to 15. The AFS combines the single tomato resonant frequency (*f* in Hz) and mass (*m*, in kg), measured by an inbuild balance, into a *FI* (firmness index, Equation (2)) [40]:

(2) $$FI=\frac{f^{2}m^{2/3}}{10^{4}}$$

### 2.5. CI Indices and Weight Loss

CI was assessed by three indices, a pitting index and uneven ripening index in MG-tomatoes, and a decay index in R tomatoes, according to Vega-García et al. [41] with slight modifications. All indices were visually assessed by the same person with the percentage of the affected tomato surface assigned to five classes (0 = no injury, 1 = < 10%, 2 = 11–25%, 3 = 26–40%, 4 = > 40%). Assessments were carried out after randomization of the samples. Tomato weight loss was expressed as the percentage weight loss over time.

### 2.6. Total Ascorbic Acid Measurement

AsA was measured according to the method by [42] with modifications. Approximately 300 mg frozen and ground tissue per tomato was extracted with 1.5 mL ice-cold 3.3% meta-phosphoric acid (MPA) and thawed on ice. The solution was vortexed for 20 s and placed in ultrasonic bath at 0 °C for 10 min in darkness. After centrifugation (25,000× *g*, 4 °C, 10 min) 1 mL extract was used for HPLC analysis of AsA. A total of 100 µL extract was mixed with 50 µL of 5 mM dithiothreitol (DTT, in 400 mM Tris base) for converting dehydroascobic acid (DHA) into AsA. After 15 min incubation in darkness and room temperature, 50 µL of 8.5% o-phosphoric acid was added into the mix to stop the reaction.

The concentration of AsA was analyzed using a HPLC equipped with a P580 pump (Dionex, Sunnyvale, CA, USA), a Dionex 340S UV-VIS detector and a MIDAS autosampler (Spark, Emmen, The Netherlands) equipped with a ProntoSIL 120-3 C18 AQ, 250 × 3 mm column (Knauer, Berlin, Germany). The column was eluted at a flow rate of 0.35 mL min^−1^ with 400 uL L^−1^ H_3_PO4 + 2.5 mL L^−1^ MeOH + 0.1 mM EDTA in miliQ followed by a wash step with 30% acetonitrile. AsA was detected at 243 nm. The system was calibrated with a standard AsA solution (Sigma Aldrich, Darmstad, Germany) prepared in 3% MPA, stabilized with 2.5 mM DTT. Total AsA concentration was calculated as the sum of measured AsA and the AsA converted from DHA.

### 2.7. Catalase (CAT) Measurement

Catalase activity was determined according to Nukuntornprakit et al. [43] with a few modifications. An extraction buffer was made with 50 mM potassium phosphate at pH 7.4, 0.1 mM EDTA and 1% Triton X-100. Five hundred milligram frozen and ground tissue per tomato was homogenized with 1 mL extraction buffer at 4 °C, then centrifuged (21,100× *g*, 4 °C, 15 min) and 0.1 mL supernatant was added to 2.9 mL of reaction mixture. The reaction mixture consisted of 50 mM potassium phosphate at pH 7.0 with 15 mM H_2_O_2_ and 0.1 mM EDTA. The decrease in absorbance was measured for 10 min at 240 nm (25 °C) with a Varian CARY 4000 spectrophotometer (Agilent, Santa Clara, CA, USA). One enzymatic unit (U) is defined as 0.01 absorbance decrease per minute and CAT activity expressed as U min^−1^ g fresh weight^−1^.

### 2.8. Hydrogen Peroxide (H_2_O_2_) Measurement

H_2_O_2_ was quantified via a colorimetric method, following Junglee et al. [44]. Briefly, 300 mg sample frozen and ground tissue per tomato was extracted in 3 mL of 0.75 mL 0.1% (*w*/*v*) trichloroacetic acid (TCA), 0.75 mL 10 mM PBS (pH 7) and 1.5 mL 1 M KI. The homogenate was centrifuged (15,000× *g*, 4 °C, 15 min) and the supernatant held for 20 min, before obtaining the absorbance at 390 nm using a Varian CARY 4000 spectrophotometer. The absorbances were converted into H_2_O_2_ concentrations based on linear calibration curve.

### 2.9. Malondialdehyde (MDA) Measurement

MDA was quantified via the thiobarbituric acid reactions (TBARS) method according to Zhang et al. (2016). Three hundred mg frozen tissue per tomato was homogenized with 0.9 mL 1% (*w*/*v*) TCA and centrifuged (10,000× *g*, 4 °C, 5 min). Later, 250 µL supernatant was mixed with 750 µL of 0.5% thiobarbituric acid in 20% TCA + 0.01% butylated hydroxytoluene. The mixture was incubated at 96 °C for 30 min and cooled in ice for 5 min followed by another centrifugation (10,000× *g*, 4 °C, 5 min) only to clarify suspension. Absorbance was determined at 532 nm and 600 nm for non-specific absorbance using an MDA molar extinction coefficient of 155 mmol L^−1^ cm^−1^ [45].

### 2.10. Statistical Analysis

Data measured at harvest were subjected to two-way ANOVA (*p* < 0.05) with either cold storage duration and light treatment or maturity and light treatment as factors. Data obtained during shelf life was subjected to mixed ANOVA, using SPSS 21 (SPSS, Chicago, IL, USA) at *p* < 0.05. Mixed ANOVA was applied with light treatment and cold duration as between subject factors and shelf-life days as within subject factor. The normality of the variables was tested applying the Shapiro–Wilk test. Mauchly’s test of sphericity was carried out to test whether variances of the differences between all possible pairs of within-subject conditions were equal. If the sphericity assumption was not fulfilled, Greenhouse–Geisser’s correction was applied to calculate the degrees of freedom. In case of a significant interactions, a pairwise comparison was carried out for each shelf-life day with LSD (least significant difference) values estimated.

## 3. Results

### 3.1. Effect of Light Treatments on CI in R and MG Fruit

**Figure 1:**
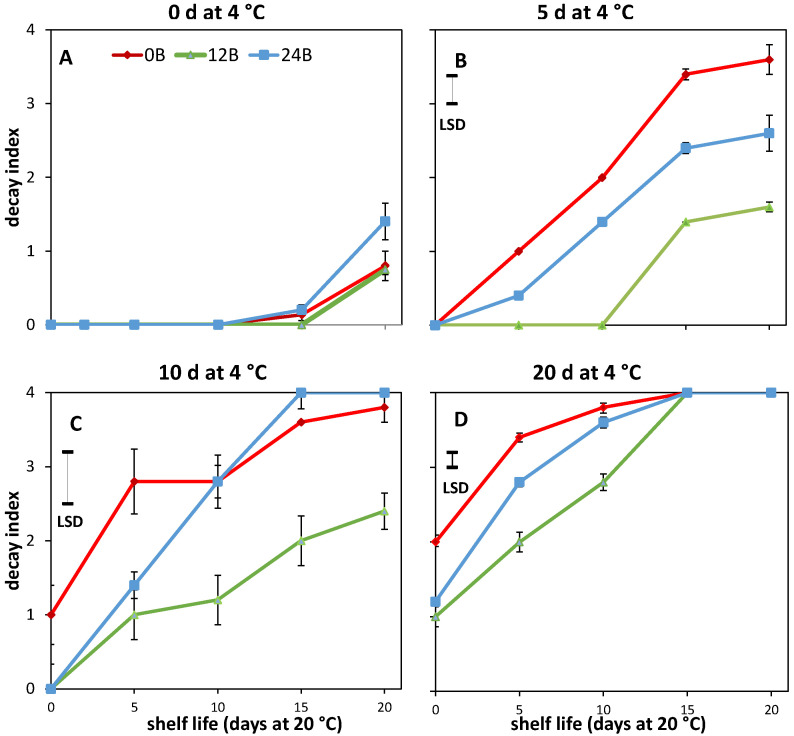
Average decay index with indicated standard error during shelf life (20 °C) for five red (R) tomatoes per cold storage duration. Red, green and blue symbols indicate cultivation at 0, 12 and 24% additional blue light, respectively. Tomatoes were either non-stored (**A**) or cold stored at 4 °C for 5 d (**B**), 10 d (**C**) or 20 d (**D**). LSD values, when present, are indicated per panel.

Increased chilling duration resulted in a higher decay index for R tomatoes at the start of shelf life (*p* < 0.001) and overall higher decay index values during shelf life (Figure 1). Severe decay occurred in R tomatoes after a shelf life of 20 d when prior being cold stored for 20 d. The decay index during shelf life for cold-stored R tomatoes was consistently lower for 12B cultivated tomatoes compared to the other light treatments (Figure 1B–D). For example, for tomatoes chilled for 5 d, the decay index of 12B was more than 74 and 140% lower than that of 24B and 0B after 10 d in shelf life, respectively. This indicates that chilling tolerance was induced for 12B cultivated R tomatoes. In MG tomatoes, chilling symptoms (pitting and uneven coloration) were observed only after 20 d of cold storage with no effects of light treatments (data not shown).

### 3.2. Light Treatments Affect the Colour and Firmness at Harvest in R Tomatoes

**Figure 2:**
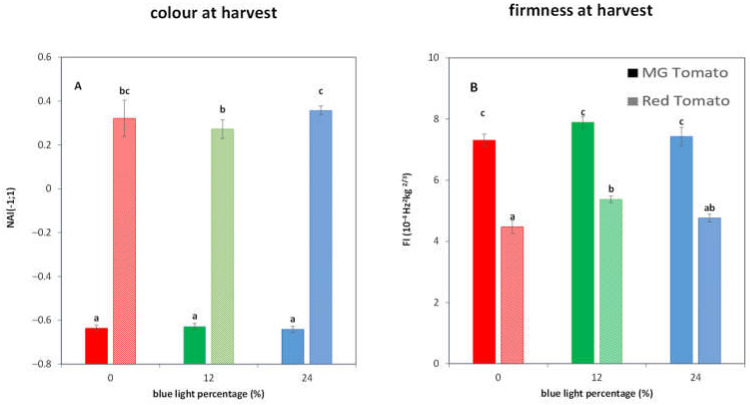
Average color at harvest (**A**), expressed as normalized anthocyanin index (NAI) and (**B**) firmness, expressed as FI index, at harvest of twenty-five MG (solid bars) and R tomatoes (dashed bars) with indicated standard error, respectively. Colors indicate tomatoes cultivated with 0 (red bars), 12 (green bars) and 24% (blue bars) additional blue light. The different letters in each panel indicate significant differences between light treatment.

At harvest, R tomatoes cultivated at 12B showed lower NAI values compared to tomatoes cultivated at 24B (Figure 2A) and higher FI index values compared to tomatoes cultivated at 0B (Figure 2B). Firmness of MG tomatoes was at least 47% higher than that of R tomatoes. The biggest difference in FI between MG and R tomatoes was found in 0B (63%) whereas 12B showed smaller difference (47%). Nevertheless, at harvest, no differences in NAI and FI values were observed for MG tomatoes with regard to the light treatments (Figure 2).

### 3.3. Effect of Light Treatments and Cold Storage on Coloration and Softening of MG Fruit

**Figure 3:**
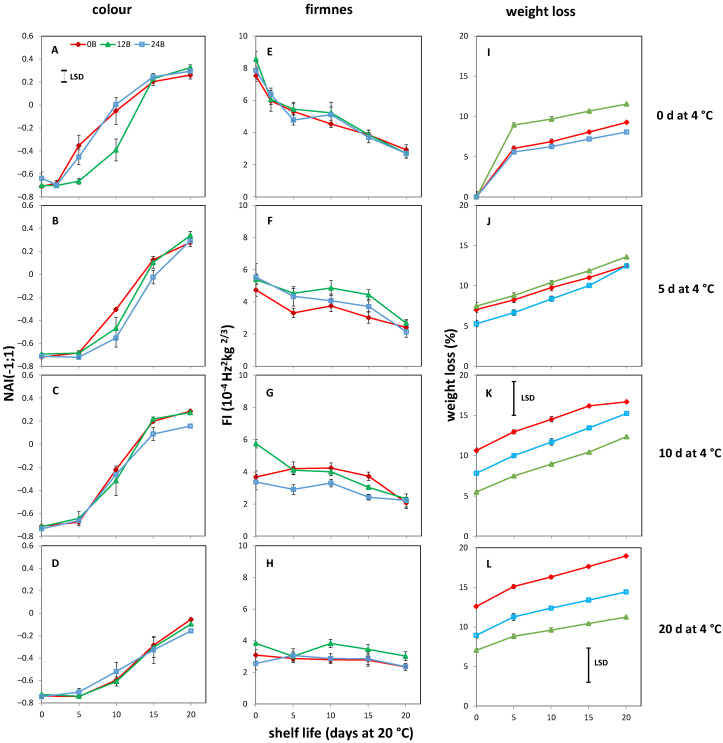
Average color, firmness and weight loss development of five MG tomatoes during shelf life at 20 °C after cold storage at 4 °C for 0 d (**A**,**E**,**I**) 5 d (**B**,**F**,**J**), 10 d (**C**,**G**,**K**) or 20 d (**D**,**H**,**L**) with indicated standard error, respectively. Colors indicate tomato cultivation with 0 (red symbols), 12 (green symbols) and 24% (blue symbols) additional blue light. LSD values, when present, are indicated per panel.

Non-chilled MG tomatoes cultivated at 12B showed a delayed increase in NAI values (Figure 3A) compared to fruit from the other light treatments, but this effect was not observed during shelf life after cold storage (Figure 3B–D). The softening of MG tomatoes was affected by the cold duration. Longer cold duration resulted in lower firmness at the start of the shelf-life period and a lower apparent softening rate during shelf life. Regardless of the cold storage duration, no effect of BL was observed on the softening of MG tomatoes during storage and during shelf life (Figure 3E–H). In MG fruit, long cold storage (10 and 20 d) resulted in lower weight loss in fruits cultivated at 12B compared to fruits of the other treatments (Figure 3K,L). Weight loss of 12B tomatoes chilled for 10 d was 94 and 43% lower than weight loss of 0B and 24B, respectively, at the start of shelf life. The differences became smaller towards the end of the shelf-life period (35 and 24%). Longer cold storage (20 d) and shelf life resulted in larger weight loss differences between 12B and the other light treatments. At the start of shelf life, the weight loss of 12B was 65 and 13% lower than that of 0B and 24B. Whereas at the end of shelf life, the weight loss of 12B tomatoes was 69 and 28% lower than the weight loss of 0B and 24B tomatoes.

### 3.4. Cold-Stored R Tomatoes Show Colour and Firmness Loss

**Figure 4:**
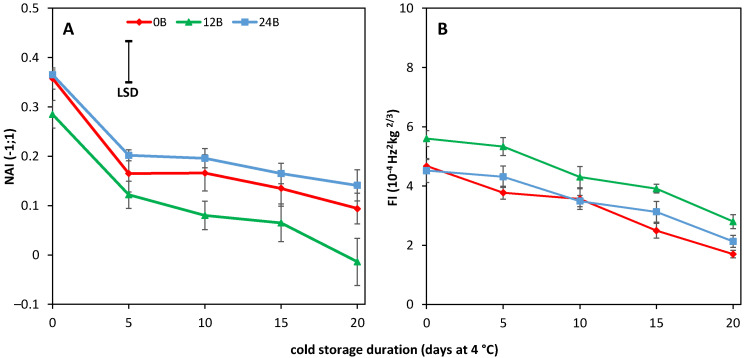
Average color development, expressed as NAI values (**A**), and average firmness development, expressed as FI index (**B**), for five red tomatoes during cold storage (4 °C), with indicated standard error. Red, green and blue symbols indicate cultivation with 0, 12 and 24% additional blue light, respectively. The LSD value in panel A indicates the presence of significant differences between light treatments.

Non chilled R tomatoes showed constant NAI values, indicating a constant red color during shelf life, irrespective of the light treatment (data not shown). Cold-stored R tomatoes showed lower NAI values and lower FI values the longer the duration of cold storage (Figure 4). The loss of red coloration was higher in R tomatoes cultivated at 12B compared to the other light treatments (Figure 4A). Firmness loss during cold storage was independent of light treatments (*p* = 0.177, Figure 4B). Longer cold storage duration resulted in lower FI values at the start of the shelf life for R tomatoes. No difference in softening rate during shelf life was observed for R tomatoes when the light treatments were compared for all cold storage durations (Figure S1).

### 3.5. AsA, CAT Activity, H_2_O_2_ and MDA Content Are Unaffected by BL Treatments

**Figure 5:**
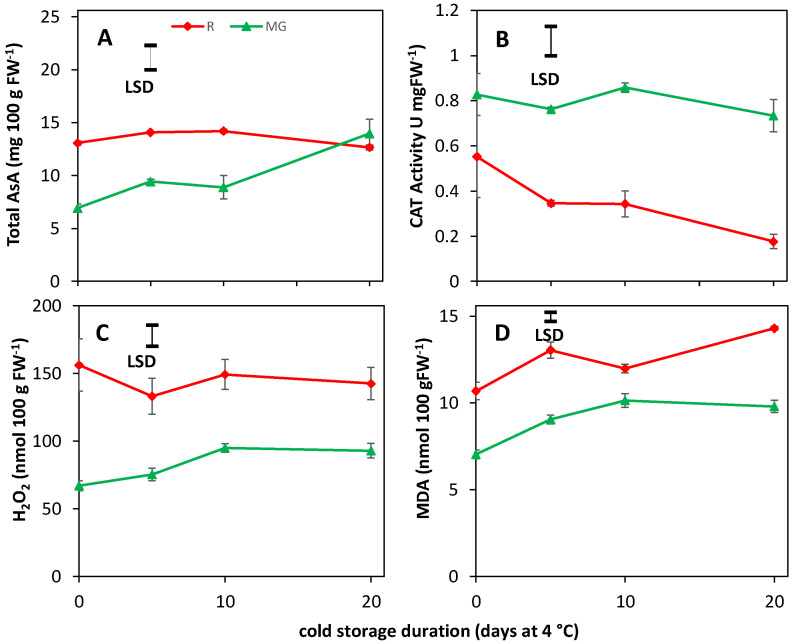
Changes in total AsA (**A**), CAT activity (**B**), H_2_O_2_ (**C**), and MDA (**D**) content at harvest and cold storage (4 °C) for fifteen MG (green symbols) and fifteen R tomatoes (red symbols). Light treatment effects were not significant and therefore values are shown only per maturity. LSD values are indicated per panel.

Total AsA, CAT activity, H_2_O_2_ and MDA contents at harvest and during cold storage were affected by the maturity at harvest. Significant differences were found between MG and R tomatoes, with lower AsA (Figure 5A) content in MG tomatoes compared R tomatoes at harvest. AsA content in MG tomatoes increased during cold storage. At the end of cold storage, MG tomatoes had a 9% higher AsA content than R tomatoes. Higher CAT activity (Figure 5B), but lower H_2_O_2_ (Figure 5C) and MDA (Figure 5D) content, was observed at harvest and during cold storage for MG compared to R tomatoes. The H_2_O_2_ production of R tomatoes did not significantly change throughout cold storage, whereas MG tomatoes showed slowly increasing H_2_O_2_ levels during cold storage. MDA levels of R and MG tomatoes showed more fluctuation in terms of difference between both maturity throughout cold storage. The differences in H_2_O_2_ fluctuated between 52, 44, 18 and 46% at 0, 5, 10 and 20 d, respectively. Despite differences with respect to maturity, levels of these compounds at harvest and during cold storage were not affected by the light treatments. This indicates that the antioxidant status as indicated by these compounds is not affected by the light treatments.

## 4. Discussion

### 4.1. Cold Tolerance Might Be Related to a Lower Lycopene Level at Harvest That Allows for More Lycopene Loss for R Tomatoes

Lycopene loss might be attributed to the chemical quenching of ^1^O_2_ or peroxyl radical by lycopene [46] or physical quenching [47]. Chemical quenching results in the breakdown of long chain carbon skeleton of lycopene at one or both fragments, which forms lycopene derivatives, such as apo-carotenals or carotenoid endoperoxides [46,47,48,49].

Red tomatoes showed a higher loss of red color when cold stored, with lower NAI values the longer the cold duration, especially for 12B cultivated tomatoes (Figure 4A). Farneti et al. [4] and Schouten et al. [39] showed that low temperature induced lycopene degradation in red ripe tomatoes could be assessed accurately by remittance spectroscopy as NAI values. There was a close relation between the NAI values and lycopene levels measured in the tomato pericarp. This might indicate that the higher cold tolerance for 12B cultivated R fruit (Figure 1) is related to a more pronounced lycopene loss during cold storage, also as no effect of light treatments were observed for AsA, CAT activity, H_2_O_2_ and MDA levels (Figure 5).

R tomatoes cultivated at 12B showed higher firmness at harvest compared to 0B (Figure 2B). Higher cold tolerance of 12B R fruit (Figure 1) could be related to the higher firmness at harvest. Increased cold tolerance for R tomatoes cultivated with additional far-red was mainly linked to higher firmness at harvest that resulted in less softening during cold storage [33]. However, light treatment effects were not observed during shelf life, regardless of the cold storage duration (Figure S1). Another option is that the higher cold tolerance for 12B cultivated fruit is linked to the lower red color at harvest (Figure 2A). This might indicate that 12B R fruits, although having lower lycopene levels at harvest, have an increased ability to lose lycopene during cold storage (Figure 4A). The scavenging activity of lycopene is reported to be inversely correlated with its concentration [50,51], which suggests that more lycopene degradation provides a higher scavenging activity, perhaps due to a higher lycopene accessibility, and thus lower CI symptoms during cold storage. The cultivation of tomatoes with increased cold tolerance through increased lycopene loss during cold storage might, however, not be desirable from both a perceived quality viewpoint [19] and a nutritional viewpoint [52].

### 4.2. Tomato Shows High Variation in Cold Tolerance Induction Pathways

Lower decay values were observed for 12B R tomatoes, but not for MG tomatoes at any of the light treatments (Figure 1). The 12B MG tomatoes might have a small increase in chilling tolerance as lower weight loss for long stored fruits was observed (Figure 3K,L) and weight loss was previously linked to chilling injury as well as membrane damage [33,53]. Nevertheless, the higher cold tolerance of R tomatoes cultivated at 12B appears mainly late during tomato development when tomatoes were already red colored. The analysis of the tomato plants cultivated in this BL experiment showed decreased biomass accumulation for 0B and 24B compared to 12B cultivated plants, likely caused by decreased photosynthetic light use (0B) or lower canopy light interception (24B) [38]. It might be hypothesized that the increased chilling tolerance for 12B cultivated tomatoes is due to increased plant biomass accumulation, but this did not result in higher antioxidant capacity indicators (Figure 5) or higher fruit dry weight percentage (data not shown).

It is likely that 0B accelerates coloring relative to 12B due to a higher portion of the red light component. Red light is reported to enhance lycopene accumulation in tomato by activating phytochromes to inhibit the accumulation of phytochrome interacting factor (PIF) proteins, which leads to increased phytoene synthase (PSY) expression [54,55,56]. On the other hand, 24B might have accelerated coloring through overexpression of cryptochrome *CRY2* by blue light irradiation, which leads to higher lycopene accumulation as [57,58,59,60,61].

Higher cold tolerance and antioxidant capacity have been linked repeatedly. In red ‘Sanibel’ tomatoes, lower CAT activity and increased MDA and AsA levels were observed after cold storage for 5 d at 5 °C [14]. In the same study, cold storage for 4 d also resulted in increased H_2_O_2_ levels, but without CI symptoms. The induction of antioxidant related defence pathways was also shown by postharvest dips [62] or induction of heat shocks proteins [63,64,65]. MG tomatoes treated with blue light during postharvest storage showed increased levels of the stress mitigator GABA and delayed coloration [66]. GABA application resulted in decreased chilling injury symptoms in loquat, anthurium, and banana [67,68,69]. This indicates multiple options to mitigate CI symptoms in tomato fruit. Our results indicate that the ability to lose lycopene or its accessibility to ROS may have increased cold tolerance. Taken together, this points to a significant variation in defence pathways that are activated in response to cold storage. Investigations into gene clusters that are activated in response to cold storage might be the way forward to understand which genotypes or treatments, either applied during cultivation or after harvest, are most suitable to mitigate CI related symptoms in tomato.

## 5. Conclusions

We hypothesized that the addition of BL during tomato cultivation induces higher antioxidant capacity to protect tomato fruit against chilling-induced oxidative stress. The chilling tolerance of red harvested tomato fruit was improved only by moderate blue light addition (12%) on top of a red background during cultivation. This improved cold tolerance for the R fruit was not due to differences in CAT activity, total ascorbic acid, H_2_O_2_ and MDA levels, but due to a lower red color at harvest and faster discoloration during cold storage. The red color measurement, measured by remittance spectroscopy, is closely related to the lycopene concentration. It is hypothesized that the lower lycopene content of the R fruit cultivated with moderate blue light levels allows for more lycopene loss during cold storage, thereby creating a higher cold tolerance.

## Data Availability

The raw data will be made available upon request.

## Supplementary Materials

The following are available online at https://www.mdpi.com/article/10.3390/biology11010101/s1, Figure S1: Average firmness index with indicated standard error during shelf life (20 °C) for five red (R) tomatoes per cold storage duration.

## References

[1] Albornoz K., Cantwell M.I., Zhang L., Beckles D.M. (2019). Integrative analysis of postharvest chilling injury in cherry tomato fruit reveals contrapuntal spatio-temporal responses to ripening and cold stress. Sci. Rep..

[2] Loayza F.E., Brecht J.K., Simonne A.H., Plotto A., Baldwin E.A., Bai J., Lon-Kan E. (2021). A brief hot-water treatment alleviates chilling injury symptoms in fresh tomatoes. J. Sci. Food Agric..

[3] Biswas P., East A.R., Brecht J.K., Hewett E.W., Heyes J.A. (2012). Intermittent warming during low temperature storage reduces tomato chilling injury. Postharvest Biol. Technol..

[4] Farneti B., Schouten R.E., Woltering E.J. (2012). Low temperature-induced lycopene degradation in red ripe tomato evaluated by remittance spectroscopy. Postharvest Biol. Technol..

[5] Sevillano L., Sanchez-Ballesta M.T., Romojaro F., Flores F.B. (2009). Physiological, hormonal and molecular mechanisms regulating chilling injury in horticultural species. Postharvest technologies applied to reduce its impact. J. Sci. Food Agric..

[6] Gill S.S., Tuteja N. (2010). Reactive oxygen species and antioxidant machinery in abiotic stress tolerance in crop plants. Plant Physiol. Biochem..

[7] Hodges D.M., Lester G.E., Munro K.D., Toivonen P.M. (2004). Oxidative stress: Importance for postharvest quality. Hortscience.

[8] Zhao R., Sheng J., Lv S., Zheng Y., Zhang J., Yu M., Shen L. (2011). Nitric oxide participates in the regulation of LeCBF1 gene expression and improves cold tolerance in harvested tomato fruit. Postharvest Biol. Technol..

[9] Biswas P., East A.R., Hewett E.W., Heyes J.A. (2016). Chilling injury in tomato fruit. Hortic. Rev..

[10] Jackman R.L., Gibson H.J., Stanley D.W. (1992). Effects of chilling on tomato fruit texture. Physiol. Plant..

[11] Marangoni A.G., Jackman R.L., Stanley D.W. (1995). Chilling-associated softening of tomato fruit is related to increased pectinmethylesterase activity. J. Food Sci..

[12] Rugkong A., McQuinn R., Giovannoni J.J., Rose J.K., Watkins C.B. (2011). Expression of ripening-related genes in cold-stored tomato fruit. Postharvest Biol. Technol..

[13] Aghdam M.S., Bodbodak S. (2014). Postharvest heat treatment for mitigation of chilling injury in fruits and vegetables. Food Bioprocess Technol..

[14] Imahori Y., Bai J., Baldwin E. (2016). Antioxidative responses of ripe tomato fruit to postharvest chilling and heating treatments. Sci. Hortic..

[15] Malacrida C., Valle E.M., Boggio S.B. (2006). Postharvest chilling induces oxidative stress response in the dwarf tomato cultivar Micro-Tom. Physiol. Plant..

[16] Hodges D.M., DeLong J.M., Forney C.F., Prange R.K. (1999). Improving the thiobarbituric acid-reactive-substances assay for estimating lipid peroxidation in plant tissues containing anthocyanin and other interfering compounds. Planta.

[17] Toor R.K., Savage G.P. (2005). Antioxidant activity in different fractions of tomatoes. Food Res. Int..

[18] Foyer C.H., Noctor G. (2011). Ascorbate and Glutathione: The heart of the redox hub. Plant Physiol..

[19] Schouten R.E., Huijben T.P., Tijskens L.M.M., van Kooten O. (2007). Modelling quality attributes of truss tomatoes: Linking colour and firmness maturity. Postharvest Biol. Technol..

[20] Heymann T., Heinz P., Glomb M.A. (2015). Lycopene Inhibits the Isomerization of β-Carotene during Quenching of Singlet Oxygen and Free Radicals. J. Agric. Food Chem..

[21] Stahl W., Sies H. (2003). Antioxidant activity of carotenoids. Mol. Asp. Med..

[22] Brandt S., Pék Z., Barna É., Lugasi A., Helyes L. (2006). Lycopene content and colour of ripening tomatoes as affected by environmental conditions. J. Sci. Food Agric..

[23] Neta-Sharir I., Isaacson T., Lurie S., Weiss D. (2005). Dual role for tomato heat shock protein 21: Protecting photosystem ii from oxidative stress and promoting color changes during fruit maturation. Plant Cell.

[24] Hernández V., Hellín P., Fenoll J., Flores P. (2015). Increased temperature produces changes in the bioactive composition of tomato, depending on its developmental stage. J. Agric. Food Chem..

[25] Lu J., Nawaz M.A., Wei N., Cheng F., Bie Z. (2020). Suboptimal temperature acclimation enhances chilling tolerance by improving photosynthetic adaptability and osmoregulation ability in watermelon. Hortic. Plant J..

[26] Jiang M.Y., Zhang J.H. (2002). Water stress-induced abscisic acid accumulation triggers the increased generation of reactive oxygenspecies and up-regulates the activities of antioxidant enzymes inmaize leaves. J. Expt. Bot..

[27] Yacoubi I., Hamdi K., Fourquet P., Bignon C., Longhi S. (2021). Structural and functional characterization of the aba-water deficit stress domain from wheat and barley: An intrinsically disordered domain behind the versatile functions of the plant abscissic acid, stress and ripening protein family. Int. J. Mol. Sci..

[28] Singh A.P., Mani B., Giri J. (2021). OsJAZ9 is involved in water-deficit stress tolerance by regulating leaf width and stomatal density in rice. Plant Physiol. Biochem..

[29] Hanin M., Brini F., Ebel C., Toda Y., Takeda S., Masmoudi K. (2011). Plant dehydrins and stress tolerance: Versatile proteins for complex mechanisms. Plant Signal. Behav..

[30] Yu Z., Wang X., Zhang L. (2018). Structural and functional dynamics of dehydrins: A plant protector protein under abiotic stress. Int. J. Mol. Sci..

[31] Hara M., Terashima S., Fukaya T., Kuboi T. (2003). Enhancement of cold tolerance and inhibition of lipid peroxidation by citrus dehydrin in transgenic tobacco. Planta.

[32] Zhuo C., Liang L., Zhao Y., Guo Z., Lu S. (2017). A cold responsive ethylene responsive factor from Medicago falcata confers cold tolerance by up-regulation of polyamine turnover, antioxidant protection, and proline accumulation. Plant Cell Environ..

[33] Affandi F.Y., Verdonk J.C., Ouzounis T., Ji Y., Woltering E.J., Schouten R.E. (2020). Far-red light during cultivation induces postharvest cold tolerance in tomato fruit. Postharvest Biol. Technol..

[34] Shi Y., Huang J., Sun T., Wang X., Zhu C., Ai Y., Gu H. (2017). The precise regulation of differentCORgenes by individual CBF transcription factors inArabidopsis thaliana. J. Integr. Plant Biol..

[35] Rihan H.Z., Al-Issawi M., Fuller M.P. (2017). Advances in physiological and molecular aspects of plant cold tolerance. J. Plant Interact..

[36] Chen B., Feder M.E., Kang L. (2018). Evolution of heat-shock protein expression underlying adaptive responses to envi-ronmental stress. Mol. Ecol..

[37] Xu F., Shi L., Chen W., Cao S., Su X., Yang Z. (2014). Effect of blue light treatment on fruit quality, antioxidant enzymes and radical-scavenging activity in strawberry fruit. Sci. Hortic..

[38] Kaiser E., Ouzounis T., Giday H., Schipper R., Heuvelink E., Marcelis L.F.M. (2019). Adding Blue to Red Supplemental Light Increases Biomass and Yield of Greenhouse-Grown Tomatoes, but Only to an Optimum. Front. Plant Sci..

[39] Schouten R.E., Farneti B., Tijskens P., Alarcón A.A., Woltering E.J. (2014). Quantifying lycopene synthesis and chlorophyll breakdown in tomato fruit using remittance VIS spectroscopy. Postharvest Biol. Technol..

[40] Schouten R.E., Fan S., Verdonk J.C., Wang Y., Kasim N.F.M., Woltering E.J., Tijskens L.M.M. (2018). Mango firmness modeling as affected by transport and ethylene treatments. Front. Plant Sci..

[41] Vega-García M.O., López-Espinoza G., Ontiveros J.C., Caro-Corrales J.J., Vargas F.D., López-Valenzuela J.A. (2010). Changes in Protein Expression Associated with Chilling Injury in Tomato Fruit. J. Am. Soc. Hortic. Sci..

[42] Davey M.W., Dekempeneer E., Keulemans J. (2003). Rocket-powered high-performance liquid chromatographic analysis of plant ascorbate and glutathione. Anal. Biochem..

[43] Nukuntornprakit O.-A., Chanjirakul K., van Doorn W.G., Siriphanich J. (2015). Chilling injury in pineapple fruit: Fatty acid composition and antioxidant metabolism. Postharvest Biol. Technol..

[44] Junglee S., Urban L., Sallanon H., Lopez-Lauri F. (2014). Optimized assay for hydrogen peroxide determination in plant tissue using potassium iodide. Am. J. Anal. Chem..

[45] Zhao D.Y., Shen L., Fan B., Yu M.M., Zheng Y., Lv S.N., Sheng J.P. (2009). Ethylene and cold participate in the regulation ofLeCBF1gene expression in postharvest tomato fruits. FEBS Lett..

[46] Edge R., Truscott T.G. (2018). Singlet Oxygen and free radical reactions of retinoids and carotenoids—A review. Antioxidants.

[47] Min D.B., Boff J.M. (2002). Chemistry and reaction of singlet oxygen in foods. Compr. Rev. Food Sci. Food Saf..

[48] Wang L., Baldwin E.A., Zhao W., Plotto A., Sun X., Wang Z., Brecht J.K., Bai J., Yu Z. (2015). Suppression of volatile production in tomato fruit exposed to chilling temperature and alleviation of chilling injury by a pre-chilling heat treatment. LWT-Food Sci. Technol..

[49] Carvalho G.C., de Camargo B.A.F., de Araújo J.T.C., Chorilli M. (2021). Lycopene: From tomato to its nutraceutical use and its association with nanotechnology. Trends Food Sci. Technol..

[50] Liu D., Shi J., Ibarra A.C., Kakuda Y., Xue S.J. (2008). The scavenging capacity and synergistic effects of lycopene, vitamin E, vitamin C, and β-carotene mixtures on the DPPH free radical. LWT-Food Sci. Technol..

[51] Kotikova Z., Lachman J., Hejtmánková A., Hejtmánková K. (2011). Determination of antioxidant activity and antioxidant content in tomato varieties and evaluation of mutual interactions between antioxidants. LWT-Food Sci. Technol..

[52] Salehi B., Sharifi-Rad R., Sharopov F., Namiesnik J., Farjadian F., Kamle M., Kumar P., Martins N., Sharifi-Rad J. (2019). Beneficial effects and potential risks of tomato consumption for human health: An overview. Nutrition.

[53] Cohen E., Shapiro B., Shalom Y., Klein J.D. (1994). Water Loss: A Nondestructive Indicator of Enhanced Cell Membrane Permeability of Chilling-injured Citrus Fruit. J. Am. Soc. Hortic. Sci..

[54] Bae G., Choi G. (2008). Decoding of light signals by plant phytochromes and their interacting proteins. Annu. Rev. Plant Biol..

[55] Casal J.J. (2013). Photoreceptor Signaling Networks in Plant Responses to Shade. Annu. Rev. Plant Biol..

[56] Leivar P., Monte E. (2014). PIFs: Systems Integrators in Plant Development. Plant Cell.

[57] Liu H., Wang Q., Liu Y., Zhao X., Imaizumi T., Somers D.E., Tobin E.M., Lin C. (2013). Arabidopsis CRY2 and ZTL mediate blue-light regulation of the transcription factor CIB1 by distinct mechanisms. Proc. Natl. Acad. Sci. USA.

[58] Zuo Z., Liu H., Liu B., Liu X., Lin C. (2011). Blue Light-Dependent Interaction of CRY2 with SPA1 Regulates COP1 activity and Floral Initiation in Arabidopsis. Curr. Biol..

[59] Xie B.X., Wei J.J., Zhang Y.T., Song S.W., Wei S.U., Sun G.W., Hao Y.W., Liu H.C. (2019). Supplemental blue and red light promote lycopene synthesis in tomato fruits. J. Integr. Agric..

[60] Alba R., Cordonnier-Pratt M.-M., Pratt L.H. (2000). Fruit-localized phytochromes regulate lycopene accumulation independently of ethylene production in tomato. Plant Physiol..

[61] Giliberto L., Perrotta G., Pallara P., Weller J.L., Fraser P.D., Bramley P.M., Fiore A., Tavazza M., Giuliano G. (2005). Manipulation of the blue light photoreceptor cryptochrome 2 in tomato affects vegetative development, flowering time, and fruit antioxidant content. Plant Physiol..

[62] Ding Y., Sheng J., Li S., Nie Y., Zhao J., Zhu Z., Wang Z., Tang X. (2015). The role of gibberellins in the mitigation of chilling injury in cherry tomato (*Solanum lycopersicum* L.) fruit. Postharvest Biol. Technol..

[63] Zhang J., Huang W., Pan Q., Liu Y. (2005). Improvement of chilling tolerance and accumulation of heat shock proteins in grape berries (Vitis vinifera cv. Jingxiu) by heat pretreatment. Postharvest Biol. Technol..

[64] Luengwilai K., Beckles D.M., Saltveit M.E. (2012). Chilling-injury of harvested tomato (*Solanum lycopersicum* L.) cv. Micro-Tom fruit is reduced by temperature pre-treatments. Postharvest Biol. Technol..

[65] Cruz-Mendívil A., López-Valenzuela J.A., Calderón-Vázquez C.L., Vega-García M.O., Reyes-Moreno C., Valdez-Ortiz A. (2015). Transcriptional changes associated with chilling tolerance and susceptibility in ‘Micro-Tom’ tomato fruit using RNA-Seq. Postharvest Biol. Technol..

[66] Dhakal R., Baek K.-H. (2014). Metabolic alternation in the accumulation of free amino acids and γ-aminobutyric acid in postharvest mature green tomatoes following irradiation with blue light. Hortic. Environ. Biotechnol..

[67] Zhang Y., Jin P., Huang Y., Shan T., Wang L., Li Y., Zheng Y. (2016). Effect of hot water combined with glycine betaine alleviates chilling injury in cold-stored loquat fruit. Postharvest Biol. Technol..

[68] Aghdam M.S., Naderi R., Sarcheshmeh M.A.A., Babalar M. (2015). Amelioration of postharvest chilling injury in anthurium cut flowers by γ-aminobutyric acid (GABA) treatments. Postharvest Biol. Technol..

[69] Wang Y., Luo Z., Huang X., Yang K., Gao S., Du R. (2014). Effect of exogenous γ-aminobutyric acid (GABA) treatment on chilling injury and antioxidant capacity in banana peel. Sci. Hortic..

