# Supplementary material for: Additional Blue LED during Cultivation Induces Cold Tolerance in Tomato Fruit but Only to an Optimum
Source: Biology (Basel). 2022 Jan 9;11(1):101. doi: 10.3390/biology11010101 (PMC8773245; doi:10.3390/biology11010101)
Supplement: Supplementary file 1 [file biology-11-00101-s001.zip › biology-1509058-supplementary.pdf]

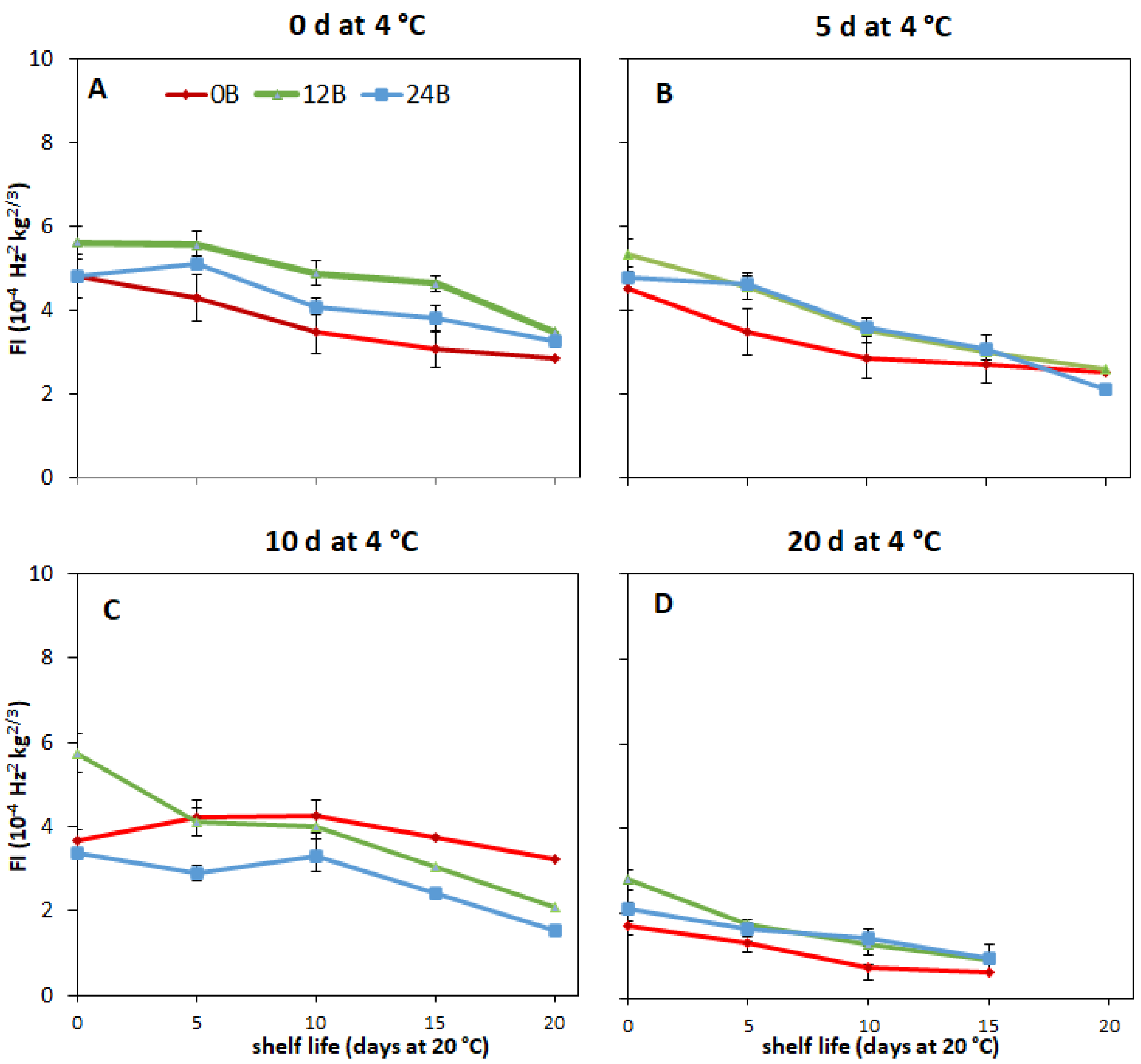

Figure S1. Average firmness index with indicated standard error during shelf life (20 °C) for five red (R) tomatoes per cold storage duration.
